# Supplementary material for: Collateral positives of COVID-19 for culturally and linguistically diverse communities in Western Sydney, Australia
Source: PLoS One. 2022 Dec 9;17(12):e0278923. doi: 10.1371/journal.pone.0278923 (PMC9733867; doi:10.1371/journal.pone.0278923)
Supplement: Attachment — Submitted filename: Response to Reviewers.docx [file pone.0278923.s003.docx]

27^th^ October 2022

*PLOS ONE*

Dear Editor,

**Re: Collateral positives of COVID-19 for culturally and linguistically diverse communities in Western Sydney, Australia**

Thank you for considering this manuscript for publication in *PLOS ONE.* This brief report presents data on the experiences of culturally and linguistically diverse communities in Western Sydney, Australia during COVID-19. We believe it is important to highlight the “positives” that CALD communities found, but more important to highlight the discrepancies between the experiences of these communities and those of the general population.

This brief report is a follow up to our previous “positives” paper which was published in the [Health Promotion Journal of Australia](https://onlinelibrary.wiley.com/doi/10.1002/hpja.494)—in which we declared in the limitations section that work needed to be done to hear from CALD communities; we have made good on that promise.

This paper has not been previously published and will not be submitted for publication elsewhere while it is under consideration at *PLOS ONE.* All named authors have agreed to the submission and have participated in the study to a sufficient extent to be named as authors. No authors declare any conflicts of interest.

I would greatly appreciate the opportunity to publish this work in PLOS ONE, a journal I believe is very suitable for this work. Similar work to ours has been published at PLOS ONE, including this [paper from New Zealand](https://journals.plos.org/plosone/article?id=10.1371/journal.pone.0249678) and [this paper from the UK](https://journals.plos.org/plosone/article?id=10.1371/journal.pone.0244873).

Thank you for reviewing the manuscript. Below we address all of the academic editor’s and reviewer’s comments.

Journal Requirements:

1. Please ensure that your manuscript meets PLOS ONE's style requirements, including those for file naming. The PLOS ONE style templates can be found at

[https://journals.plos.org/plosone/s/file?id=wjVg/PLOSOne_formatting_sample_main_body.pdf](https://protect-au.mimecast.com/s/xoZ-CE8wmrtWVpQ8MiPD5Hh?domain=journals.plos.org) and

[https://journals.plos.org/plosone/s/file?id=ba62/PLOSOne_formatting_sample_title_authors_affiliations.pdf](https://protect-au.mimecast.com/s/6p64CGv0oyCJKAVNGsk39b3?domain=journals.plos.org)

**RESPONSE:** We have checked to make sure our manuscript conforms to these requirements.

2. Please provide additional details regarding participant consent. In the ethics statement in the Methods and online submission information, please ensure that you have specified (1) whether consent was informed and (2) what type you obtained (for instance, written or verbal, and if verbal, how it was documented and witnessed). If your study included minors, state whether you obtained consent from parents or guardians. If the need for consent was waived by the ethics committee, please include this information.

If you are reporting a retrospective study of medical records or archived samples, please ensure that you have discussed whether all data were fully anonymized before you accessed them and/or whether the IRB or ethics committee waived the requirement for informed consent. If patients provided informed written consent to have data from their medical records used in research, please include this information.

**RESPONSE:** Thank you. We have now provided additional changes in the manuscript which details participant consent. The participant consent and recruitment methods now reads;

*This study involves human participants and was approved by the Western Sydney Local Health District Human Research Ethics Committee (Project number 2020/ETH03085). Participants gave informed consent to participate in the study before taking part.*

*Participants were eligible to take part if they were aged 18 years or over and spoke one of the following as their main language at home: Arabic, Assyrian, Chinese, Croatian,*

*Dari, Dinka, Hindi, Khmer, Samoan, Tongan, Spanish. Through iterative discussions with Multicultural Health and Health Care Interpreter Service staff in each partic- ipating Local Health District, we selected 11 language groups that would provide broad coverage across different global regions, and groups with varying average levels of English language proficiency (based on 2016 Australian census data28), varying access to translated materials and varying degrees of reading skill in their main language spoken at home.*

*Participants were recruited through bilingual Multicultural Health staff and Health Care Interpreter Service staff. Multicultural Health staff recruited participants through their existing networks, community events and community champions. Health Care Interpreter Service staff recruited participants at the end of a medical appointment and via their community network. The survey was hosted online using the web-based survey platform Qualtrics. Potential participants were offered two means of taking part: completing the survey them- selves online (available in English or translated), or with assistance from bilingual staff or an interpreter who read the questions to them and recorded their responses. To ensure consistency in the phrases used for assisted survey completion, translated versions of the survey were provided to all staff assisting with survey completion. Translations were completed by translators with National Accreditation Authority for Translators and Interpreters (NAATI) accreditation where possible (12).*

3. Thank you for stating the following financial disclosure:

"This study was not specifically funded. "

At this time, please address the following queries:

a)        Please clarify the sources of funding (financial or material support) for your study. List the grants or organizations that supported your study, including funding received from your institution.

b)        State what role the funders took in the study. If the funders had no role in your study, please state: “The funders had no role in study design, data collection and analysis, decision to publish, or preparation of the manuscript.”

c)        If any authors received a salary from any of your funders, please state which authors and which funders.

d)        If you did not receive any funding for this study, please state: “The authors received no specific funding for this work.”

Please include your amended statements within your cover letter; we will change the online submission form on your behalf.

**RESPONSE:** Thank you. We choose option d).

*The authors received no specific funding for this work.*

4. We note that you have stated that you will provide repository information for your data at acceptance. Should your manuscript be accepted for publication, we will hold it until you provide the relevant accession numbers or DOIs necessary to access your data. If you wish to make changes to your Data Availability statement, please describe these changes in your cover letter and we will update your Data Availability statement to reflect the information you provide.

**RESPONSE:** We would like to update our data sharing arrangement to **‘Data available upon reasonable request’.**

This is due to the sensitive nature of this data and the culturally and linguistically diverse communities we have been working with, and furthermore because of the involvement of the Western Sydney Local Health District as key stakeholders.

5. Please review your reference list to ensure that it is complete and correct. If you have cited papers that have been retracted, please include the rationale for doing so in the manuscript text, or remove these references and replace them with relevant current references. Any changes to the reference list should be mentioned in the rebuttal letter that accompanies your revised manuscript. If you need to cite a retracted article, indicate the article’s retracted status in the References list and also include a citation and full reference for the retraction notice.

**RESPONSE:** Thank you. We have reviewed the reference list to make sure that all included references are up-to-date and conform the PLOS ONE standards.

Additional Editors Comments:

Please address the concerns/questions below:

1) Full name of the Institutional Review Board (IRB) or the Ethics Committee that authorized the study

**RESPONSE:** Thank you. We have updated this in the manuscript. It now reads

*This study involves human participants and was approved by the Western Sydney Local Health District Human Research Ethics Committee (Project number 2020/ETH03085).*

2) Outline the details of the recruitment of study participants here instead of referring potential readers to a prior publication

**RESPONSE:** Thank you. We have included details of participant recruitment rather than directing to our related manuscript. It now reads;

*Participants were recruited through bilingual Multicultural Health staff and Health Care Interpreter Service staff. Multicultural Health staff recruited participants through their existing networks, community events and community champions. Health Care Interpreter Service staff recruited participants at the end of a medical appointment and via their community network. The survey was hosted online using the web-based survey platform Qualtrics. Potential participants were offered two means of taking part: completing the survey them- selves online (available in English or translated), or with assistance from bilingual staff or an interpreter who read the questions to them and recorded their responses. To ensure consistency in the phrases used for assisted survey completion, translated versions of the survey were provided to all staff assisting with survey completion. Translations were completed by translators with National Accreditation Authority for Translators and Interpreters (NAATI) accreditation where possible (12).*

3) What is the single item health literacy screener used in this study?

**RESPONSE:** Thank you. We have included details of the single item health literacy screener in the manuscript.

*The single item health literacy screener is ‘How confident are you filling out medical forms by yourself?’*

Reviewer comments

Reviewer #1: I would have liked to see greater depth to your survey and additional questions that sought to refute or further analyze the areas identified as positives from other studies. For example, what aspects of family time improved, further differentiation between self care aspects like diet or exercise or mental wellbeing. Is there a greater understanding of whether the relationship enhancement occurred more with family or with friends. When money was spent less on certain elements was it invested in different ways, was debts reduced? How was gratitude expressed to other? In paying attention to those in need, how was that quantified? I would liked to have seen greater emphasis of statistically significant categories and an attempt to hypothesis why findings were identified.

**RESPONSE:** We thank the reviewer for these thoughtful ideas. We fully agree that these ideas are worth exploring. Unfortunately, these ideas did not fall in the scope of our study. This manuscript presents the findings from a small part of a larger piece of work which has been published elsewhere and is referred to in the manuscript. This manuscript is intended to be a brief report only, and reporting only the ‘positives’ item from our original survey which gave rise to much larger pieces of work which are referenced in the manuscript.

Kind regards,

Samuel Cornell, on behalf of all authors
